# No long-term impact of low-energy distal radius fracture on health-related quality of life and global quality of life: a case-control study

**DOI:** 10.1186/1471-2474-10-106

**Published:** 2009-08-25

**Authors:** Gudrun Rohde, Glenn Haugeberg, Anne Marit Mengshoel, Torbjorn Moum, Astrid K Wahl

**Affiliations:** 1 Department of Rheumatology, Sorlandet Hospital, Kristiansand, Servicebox 416, 4604 Kristiansand, Norway; 2 Institute of Nursing and Health Sciences, Medical Faculty, University of Oslo, Pb.1153 Blindern, 0316 Oslo, Norway; 3 Department of Behavioural Sciences in Medicine, Medical Faculty, University of Oslo 1111, Blindern, 0317 Oslo, Norway; 4 Centre for Shared Decision Making and Nursing Research Rikshospitalet, N-0027 Oslo, Norway

## Abstract

**Background:**

Changes in patient-reported outcomes like health related quality of life (HRQOL) and global quality of life (GQOL) in patients with low-energy distal radius fracture might be related to fracture, or be within the normal range of variation in an elderly population. Hence, the present study aims to examine: Whether patients with low-energy distal radius fracture attain their pre-fracture levels in HRQOL and GQOL one year after the fracture and compare these levels with age- and sex-matched controls; and whether objective factors predict changes in HRQOL and GQOL during the same one year period.

**Methods:**

We examined 160 patients and 169 age- and sex matched controls, respectively (mean ± SD) 67 ± 9 and 66 ± 9 years of age. HRQOL was assessed by the Modified Health Assessment Questionnaire (MHAQ) and the Short–Form 36 (SF-36). The Quality of Life Scale (QOLS) assessed GQOL. Paired sample t-tests and multiple linear regression analyses were applied.

**Results:**

After one year no differences were found in HRQOL (assessed as arm functions, physical health and mental health) compared to pre-fracture level in the patient group. Both patients with distal radius fracture and controls reported a reduced GQOL after one year (p < 0.001). Low-energy distal radius fracture did not predict worsened HRQOL or GQOL one year after inclusion, and few predictors of changes were identified. Worsened arm function was predicted by low BMI (B = -0.20, p = 0.019) at baseline, worsened physical health was predicted by low education (B = 1.37, p = 0.017) at baseline, and living with someone predicted worsened mental health (B = 2.85, p = 0.009)

**Conclusion:**

Patients with a distal radius fracture seem to manage well despite the fracture, and distal radius fracture is not an independent predictor of worsened HRQOL and GQOL.

## Background

The distal radius is a frequent site of osteoporotic fractures in elderly and seems to occur most frequently among relatively healthy elderly people [1,2]. Distal radius fractures do also occur about 15 years earlier in life than other osteoporotic fractures like hip fractures [3,4]. Furthermore, a low-energy distal radius fracture has been identified as a predictor of future fracture of both hip and spine [4]. Patients with low-energy distal radius fractures report reduced arm functions and pain the first weeks after the fracture, and some patients may never regain pre-fracture arm functions [5-7]. This may impact quality of life (QOL).

QOL cover different physical, psychological and social aspects, and emphasize the patients' perception of these aspects. QOL comprises both health-related quality of life (HRQOL) defined as an individuals' experience of their general state of health, such as physical, social, and mental well-being [8] and global quality of life (GQOL) reflecting an individuals' satisfaction with life, and has a meaning beyond an individuals' health [9].

To identify changes in subjective outcomes such as HRQOL and GQOL after a fracture might give patients and their caregivers a better understanding of expected recovery. Previous studies of HRQOL after distal radius fractures have shown that most recovery in arm functions occurs during the first 6 months after the fracture, and at one year follow-up most patients report no or minimal pain and disability [6,10-12]. Furthermore, patients with a distal radius fracture seem to reach population-based levels of HRQOL some time after the fracture, although numerous patients report remaining symptoms from the fracture [5,7]. Education, co-morbidities and injury compensation at baseline seem to be covariates of how patients report their pain and disability one year after a distal radius fracture, indicating that also factors independent of the injury play a role in self-reported arm functions after the fracture [13]. In other studies, low bone mineral density (BMD) and low body mass index (BMI) are identified as determinants of reduced HRQOL two years after a distal radius fracture [7].

Previous research on patients with low-energy fracture seems to lack a broader perspective in one and the same study, including both objective factors, such as BMD, BMI and other demographic and clinical measures, as well as patient-reported outcome like HRQOL and GQOL. Presently, all these variables are assessed. Furthermore, we ask whether the changes in HRQOL and GQOL in patients with low-energy distal radius fracture are related to the fracture or within the normal range of variation in an elderly population [14]. Hence, the present study aims to examine:

1) Whether patients with low-energy distal radius fracture attain their pre-fracture levels in HRQOL and GQOL one year after the fracture, and compare these levels with age- and sex-matched controls;

2) Whether objective factors predict changes in HRQOL and GQOL during the same one year period.

## Methods

### Study design, patients and controls

To study one-year changes in HRQOL and GQOL in patients with low-energy distal radius fracture we applied a case-control, prospective longitudinal study design. The study was recommended by the Regional Committee for Medical Research Ethics and approved by the National Data Inspectorate.

Patients with low-energy distal radius fracture aged 50 years and older were consecutively recruited from an osteoporosis centre at a regional hospital in southern Norway in 2004 and 2005. A low-energy fracture was defined as a minimal trauma falling from standing height or less [15], and a distal radius fracture was defined as located within 3 cm of the radio-carpal joint [16]. The distal radius fractures were closed injuries, and the fractures were treated conservatively by stabilising the fracture by a plaster cast or by external fixation. Patients were assessed and data collected in median 10 days (interquartile range 13) after fracture and reassessed one year after fracture. With regard to demographical and clinical variables, HRQOL and GQOL the patients were asked to report their status prior to fracture. The patients also were asked to report their exercise habits, falls and the use of health care recourses during the year before fracture. The controls were asked about their status and habits prior to inclusion. The same data collection performed at baseline was repeated after one year.

The included patients comprised 56% of all 324 patients with low-energy distal radius fracture treated at the hospital, and 73% of 249 patients examined at the osteoporosis centre. Before inclusion in this study, we confirmed that the fracture was not a result of high-energy trauma and was caused only by minimal trauma according to the definition of low-energy fracture [17]. Patients who were excluded comprised a total of 51 patients with confusion or dementia, serious infection, patients not capable of giving informed consent, patients not capable of speaking Norwegian or tourists and 92 patients who did not want to participate in the study.

At baseline 181 patients with distal radius fracture were included along with 181 age- and sex-matched controls. The age- and sex-matched controls were randomly allocated from the national registry for the catchment area and invited by mail to participate in the study. The controls were identified consecutively along with patient recruitement. If a potential control refused to participate or did not respond to the invitation, a new control was invited. Overall, 131 potential controls refused to participate or did not respond to the invitation. We aimed an age match of ± 1 year in the patients with distal radius fracture; however, this was a challenge for some of the patients aged 80 years and older. In these patients we accepted a match of ± 5 years, except for one woman aged 96 years who was matched with an 86 years old control.

### Demographical and clinical variables

Demographical and clinical data (listed in table 1) were collected, and included also exercise, smoking habits, medication, previous fracture, number of falls the year before the fracture, and co-morbidity. Furthermore, patients and controls reported their use of health care resources; like visiting general practitioners, medical specialists, physiotherapists, and hospitalization the year prior to the fracture or prior to inclusion in the control group. Regular exercise was defined as walking or doing more intensive exercise more than 30 minutes three times a week. Previous fracture was defined as a low-energy trauma fracture after the age of 50. Co-morbidity included heart diseases, pulmonary diseases, neurological disorders, urogenital disorders, gastrointestinal disorders, endocrine disorders, inflammatory joint disorders and connective tissue disorders, cancer, mental disorders. For co-morbidity, we also computed a sum score of the number of diseases in each patient and control, which was used in the multivariate analyses.

**Table 1:** Baseline demographical and clinical characteristics in patients with low-energy distal radius fracture and controls who visited the osteoporosis centre at both inclusion and at one year follow up.

	**Distal radius fracture**	**Controls**	p*
	n = 160	n = 169	
**Demographics**			
Age (years; mean (SD))	67 (9)	66 (9)	0.457
Females	144 (90)	151 (89)	0.846
BMI (kg/m^2^)	25.7 (4.3)	26.7 (4.3)	**0.027**
Menarche (years; mean (SD))	13.9 (1.5)	13.6 (1.4)	0.066
Menopause (years; mean (SD))	48.9 (4.5)	49.6 (4.1)	0.086
Education			**0.011**
< 10 years	56 (38)	70 (42)	
11–13 years	61 (42)	45 (27)	
> 13 years	30 (20)	53 (31)	
Co-habiting	84(53)	112 (67)	**0.011**
Regular exercise**	119 (74)	126 (74)	0.970
Current smoker	23 (14)	21 (12)	0.604
**Clinical characteristics**			
Current calcium and/or vitamin D treatment	39 (24)	41 (24)	0.981
Current ART	28 (18)	22 (13)	0.258
Previous fractures	83 (52)	77 (47)	0.319
≥ 1 fall in the previous year	68 (47)	48 (36)	0.054
Osteoporosis	52 (32)	30 (18)	**<0.001**
Osteopenia	83 (52)	74 (44)	
Normal BMD	25 (16)	64 (38)	
Heart diseases	48 (30)	58 (34)	0.402
Pulmonary diseases	19 (12)	12 (7)	0.138
Neurological diseases	12 (8)	14 (8)	0.792
Endocrine disorders	14 (9)	20 (12)	0.358
Gastrointestinal disorders	8 (5)	21 (12)	**0.018**
Urogenital disorders	5 (3)	1 (1)	0.086
Inflammatory joint disorders and connective tissue disorders	36 (23)	45 (26)	0.385
Cancer	16 (10)	19 (11)	0.715
Mental disorders	7 (4)	11 (7)	0.395
Co-morbidities (range 0–6)	1.0 (1.0)	1.2 (1.1)	0.191

Mean (SD) for continuous variables and numbers (%) for categorical variables.

*Bold p-values indicate significant differences between the groups

** Exercise more than 30 minutes three times a week.

BMI, body mass index; ART, antiresorptive treatment, a specific osteoporosis treatment comprising biphosphonates, or selective oestrogen-receptor modulators.

### Bone density measurements

Standardized BMD measurements at lumbar spine L2-4 and both hips were performed by four trained nurses using the same dual energy X-ray absorptiometry (DXA) equipment (General Electric, Lunar Prodigy) at baseline and at one year follow-up. The machine was stable over the entire measurement period. Long term spine phantom in-vitro coefficient of variation (CV) for the whole period was 0.62%. The in-vivo CV for the measurement procedure was 1.19% at lumbar spine L2-4, 0.95% at right total hip and 0.89% at left total hip. The BMD measurements were expressed as T-scores (SD) calculated on the basis of the reference value in the DXA machine provided by the manufacturer. Osteoporosis was defined as T-score ≤ -2.5 SD, osteopeniae as T-score > -2.5 and < -1.0 and normal BMD as T-score > -1.0, according to the WHO definition for osteoporosis [17].

### Modified Health Assessment Questionnaire (MHAQ)

Modified Health Assessment Questionnaire (MHAQ) measures a patients ability to perform activities of daily living [18,19]. Although primarily developed as a measure for use in rheumatoid arthritis, MHAQ has been used across a variety of diseases [20]. The MHAQ consists of 8 items covering daily activities including skills that demand a good arm function e.g. dressing, lift a full cup or glass to the mouth, wash and dry the entire body [18,19]. The total mean scores range from 1–4, with 1 representing "without any difficulty". For incomplete questionnaires, the missing values were replaced with the mean value of the answered questions of the respondent when at least 6 out of 8 items had valid response, which is based on the scale instructions given by the developers of the questionnair [20,21]. At baseline all the the patients and controls had valid responses. At one year follow-up 1,5% of the patients and 1% of the controls had one or two missing responses. In the multivariate analyses, which were performed to identify if a low-energy distal radius fracture was a predictor of worsened arm functions, we rescaled MHAQ from 0 to 100, with 100 representing "without any difficulty" in accordance with prior studies [21,22].

### Short Form – 36 (SF-36)

The Short- Form 36 (SF-36) was used to assess HRQOL (physical and mental health) [23,24]. The SF-36 includes eight domains (general health, bodily pain, physical functioning, physical role limitations, mental health, vitality, social functioning, and emotional role limitations), which can be combined into a physical health summary scale and a mental health summary scale. The physical component summary (PCS) and mental component summary (MCS) scales were used in this study. The SF-36 scales were scored according to published scoring procedures, and each was expressed as a value from 0 to 100, with 100 representing "excellent health". For incomplete questionnaires substitution of missing values is based on the scale instructions given by the developers of the questionnaire [23,24]. At baseline 5.6% of the patients and 13.5% of the controls had one or more missing responses. At one year follow-up 18.8% of the patients and 14% of the controls had one or more missing responses. The questionnaire has been thoroughly tested for psychometric properties in other studies, within several countries, including Norway [23-26].

### Quality of Life Scale (QOLS)

The Quality of Life Scale (QOLS), a 16-item, domain-specific instrument adapted by Burckhardt et al. for people with chronic conditions, was used to assess GQOL [9,27,28]. In this questionnaire GQOL is understood as a broad range of human experiences related to one's overall well-being and satisfaction. The QOLS is a self-administered questionnaire [27,29]. The items are rated at a 7-point satisfaction scale. For incomplete questionnaires, the missing values were replaced with the mean value of the answered questions of the respondent when at least 80% of the items had a valid response. The substitution of missing values is based on the scale instructions given by the developers of the questionnaire [9,27]. At baseline 26% of the patients and 23% of the controls had one or more missing responses. At one year follow-up 35% of the patients and 33% of the controls had one or more missing responses. The items with most missing responses were QOLS item number four (having and rearing children) and item five (close relationship with spouse or other significant other).

The questionnaire is scored by adding up the items to obtain a total score from a minimum of 16 to a maximum of 112. Higher scores indicate better GQOL. Burckhardt et al. [28] suggested that the QOLS comprising three sub-dimensions: relationship and marital well-being (items 3, 4, 5, 6, and 14); health and functioning (items 1, 2, 11, 15, and 16); and personal, social, and community commitment (items 7, 8, 9, 10, 12, and 13) [28,30]. The three dimensions are scored by summing the scores for each item in the dimension. The questionnaire has been thoroughly tested for psychometric properties in other studies, within several countries [28,30-32].

### Statistical analysis

Statistical analyses were carried out using the Statistical Package for Social Sciences (SPSS) for Windows (version 16.0). Chi-square tests and t-tests were used to compare differences between subgroups. Wilcoxon rank tests were used to compare continuous health care resources data between inclusion and one year follow-up, and paired samples t-tests were used to compare HRQOL and GQOL at inclusion and one year follow-up within the patients with distal radius fracture and within the controls. Furthermore, standard difference scores (s-scores) were calculated by subtracting the mean MHAQ, SF-36 or QOLS scores at baseline from the mean score of one year follow-up, and then dividing by the standard deviation (SD) at baseline [33]. To estimate the proportion of patients and controls with clinically significant changes in HRQOL and GQOL, we also identified participants with modest changes (between -5 and -10%), moderate changes (between -10 and -20%) and substantial changes (more than -20%) between baseline and one year follow-up [33,34]

Multiple linear regression analyses (procedure GLM in the SPSS) were used to identify significant predictors of worsened HRQOL (delta total mean MHAQ, SF-36; delta PCS and delta MCS) or GQOL (delta QOLS) in the study-population (both patients and controls). The regression analyses were adjusted for baseline total mean MHAQ (rescaled), PCS, MCS or QOLS respectively, at inclusion. The independent variables in the multiple regression analyses were selected based on results from earlier studies which show that age, sex, education level, marital status, BMD, falls, BMI, co-morbidity and osteoporotic fractures appear to be associated with HRQOL and/or GQOL, and these variables were all included in the regression model [14]. To test if the effects of predictors of change in our dependent variables were significantly different for patients and controls, interaction terms involving the patient/control dichotomy and each of the predictors were entered one pair at a time, while retaining main effects in the model. The level of significance was set at 0.05.

## Results

### Respondents

The patients in the study were significantly (p < 0.001) younger (67 ± 9 years) than the excluded patients (76 ± 12 years) and those who did not want to participate (72 ± 11 years). Among the 181 patients with distal radius fracture and 181 controls included at baseline, 160 patients and 169 controls attended the osteoporosis-centre at one year follow-up. There were minor differences between participants at one year follow-up and participants who were lost to follow-up. Among patients with distal radius fracture a statistically significant difference was only reported for gastrointestinal disorders (p = 0.015). Among controls, those who were lost to follow-up were significantly older (p = 0.046), and reported significantly lower SF-36 score in vitality (p = 0.014).

### Demographical and clinical characteristics and use of health care resources

Socio-demographical and clinical characteristics at baseline of those participants who completed both baseline and one year follow-up assessments are shown in table 1.

Patients with distal radius fracture more often were living alone (p = 0.011), had fewer years of education (p = 0.011), had lower BMI (p = 0.027), and were more frequently classified with osteoporosis (p < 0.001) compared to the controls. The distal radius fracture occurred indoors in 31 (19%) patients and outdoors in 129 patients (81%). Mean age in patients whose fracture occurred indoors was 70 ± 11 years old and outdoors 66 ± 9 years old (p = 0.074).

At one year follow-up, the patients with distal radius fracture compared with controls were also more frequent user of calcium and/or vitamin D treatment (103 vs. 88, p = 0.024) and antiresorbtive treatment (ART) (52 vs. 27, p < 0.001). Four patients with distal radius fracture and no controls got a new fracture between inclusion and one-year follow-up (p = 0.069).

During the one year follow-up, patients with distal radius fracture on average visited their general practitioner more frequently than the year before fracture (3.9 vs. 3.4, p = 0.006). There were no significant differences with regard to the number of visits to other health care providers, like medical specialists (p = 0.083), physiotherapist (p = 0.139) or number of days hospitalized the last year (p = 0.581), the year following the fracture compared to number of visits the year before fracture. There were no significant changes in the controls.

### Changes in HRQOL and GQOL

No significant changes in arm function as assessed by MHAQ were identified in patients with distal radius fracture at one year follow-up compared to the baseline (prior to fracture) assessment (p = 0.202) (table 2). Only ten patients (6%) with a distal radius fracture did not attain their pre-fracture arm functions one year after fracture.

**Table 2:** Health-related quality of life in patients with low-energy distal radius fracture and controls at baseline and after one year.

	**Patients with distal radius fracture (n = 160)**					**Controls (n = 169)**				
	**Baseline**	**One year**	**p**	**Effect size**	**Mean change (SD)**	**Baseline**	**One year**	**p**	**Effect size**	**Mean change (SD)**

MHAQ*	1.04 (0.16)	1.05 (0.21)	0.202	0.06	0.01 (0.1)	1.06 (0.22)	1.06 (0.20)	0.802	0.01	0.0 (0.2)
SF-36 **										
PCS	51.2 (9.4)	50.4 (9.8)	0.209	-0.1	-0.8 (7.6)	51.2 (8.4)	51.3 (8.6)	0.846	0.1	0.1 (5.9)
MCS	50.2 (9.9)	50.3 (10.5)	0.840	0.01	0.2 (9.2)	51.7 (8.4)	51.7 (8.6)	0.908	0.1	0.1 (7.2)

Data are given as means with standard deviation, and paired sample t-tests were applied to detect significant differences between baseline and follow-up.

* the MHAQ scores range from 1 to 4, where 1 means high perception of their ability to perform activities of daily living.

** The score for SF-36 ranges from 0 to 100, where 100 means high HRQOL.

PCS = physical component summary, MCS = mental component summary, MHAQ = The Modified Health Assessment Questionnaire.

Furthermore, no significant changes were identified in HRQOL as assessed by the SF-36; physical health (p = 0.209) and mental health (p = 0.840) from pre-fracture to one year after the fracture in the patients with a distal radius fracture. The same pattern was seen in controls (table 2).

With regard to GQOL, the patients with distal radius fracture reported significantly lower total GQOL score (p < 0.001, s-score = -0.4) and for the sub-dimensions; relationship and marital well-being (p = 0.015, s-score = -0.2), health and functioning (p = 0.001, s-score = -0.2) and personal, social and community commitment (p < 0.001, s-score = -0.3) at one year follow-up compared to the baseline assessment (table 3). In the controls we also found significant changes in GQOL scores within both the overall score and the three sub-dimensions (p < 0.001) one year after inclusion, with s-score = -0.6 in QOLS, s-score = -0.3 for relationship and marital well being, s-score = -0.4, s-score = -0.4 for health and functioning and s-score = -0.6 in personal, social and community commitment (table 3).

**Table 3:** Global quality of life in the patients with low-energy distal radius fracture and controls at baseline and after one year.

	**Patients with distal radius fracture (n = 160)**					**Controls (n = 169)**				
	**Baseline**	**After one year**	P****	**Effect size**	**Mean** ** change(SD)**	**Baseline**	**After one year**	P****	**Effect size**	**Mean** ** change(SD)**

Total QOLS-score *	94.4 (10.5)	90.8 (12.6)	**<0.001**	-0.4	-4.0 (8.9)	97.3 (8.4)	92.7 (10.2)	**<0.001**	-0.4	-4.9 (7.8)
Relationship and Marital Well-being**	31.5 (3.0)	30.9 (3.2)	**0.015**	-0.2	-0.7 (3.0)	32.1 (2.9)	31.1 (3.0)	**0.015**	-0.2	-1.0 (2.4)
Health and Functioning**	29.1 (3.9)	28.2 (4.5)	**0.001**	-0.2	-0.9 (3.2)	30.0 (3.4)	28.7 (4.0)	**0.001**	-0.2	-1.3 (3.1)
Personal, Social and Community Commitment***	34.0 (5.0)	32.3 (5.8)	**<0.001**	-0.3	-1.8 (4.7)	35.2 (3.8)	33.0 (5.0)	**<0.001**	-0.3	-2.1 (4.6)

Data are given as means with standard deviation, and paired sample t-tests were applied to detect significant differences between baseline and follow-up.

* Range from 16 to 112, where 112 means high GQOL.

**Range 5–35, where 35 means high GQOL.

***Range 6–42, where 42 means high GQOL.

**** P-values marked with bold indicate statistically significant p-values.

QOLS = quality of life scale.

Modest (-5 to -10) or moderate (-10 to -20) worsening arm functions between baseline and one year follow up were reported by 2.5% of the patients and 3% of the controls and substantial change (-20 or more) in 2 patients and one control. Modest or moderate worsening of physical health was reported by 20% of the patients and 10% of the controls, and substantial changes in one patient. Modest or moderate worsening of mental health was reported by 19% of the patients and 14% of the controls and substantial changes in four patients and in one control. Furthermore, modest or moderate worsening of GQOL was reported by 35% of the patients and 42% of the controls.

No significant differences between the patients with distal radius fracture and controls were identified in HRQOL and GQOL at one year follow-up.

### Prediction of changes in HRQOL and GQOL

A low-energy distal radius fracture did not predict worsened HRQOL or GQOL one year after inclusion, and few predictors of changes were identified. Worsened arm function was predicted by low BMI (B = -0.20, p = 0.019) at baseline, worsened physical health was predicted by low education (B = 1.37, p = 0.017) at baseline, and living with someone predicted worsened mental health (B = 2.85, p = 0.009) (table 4).

**Table 4:** Predictors of change in health-related quality of life (delta MHAQ, delta PCS, and delta MCS) and global quality of life (delta QOLS) in both patients with low-energy distal radius fracture (n = 160) and controls (n = 169).

	**MHAQ Adj B (95% CI)**	**p**	**PCS Adj B (95% CI)**	**p**	**MCS Adj B (95% CI)**	**p**	**QOLS Adj B (95% CI)**	**P**
**Demographic**								
Age*	-0.06	0.878	-0.90	0.101	-0.09	0.885	-0.48	0.520
	(-0.90, 0.77)		(-1.97, 0.18)		(-0.14, 0.12)		(-1.74, 0.88)	
Male	-0.24	0.840	0.03	0.984	-0.77	0.662	-2.16	0.222
	(-2.56, 2.08)		(-2.87, 2.93)		(-4.24, 2.70)		(-5.62, 1.32)	
Female	Ref		Ref		Ref		Ref	
Education	0.83	0.060	1.37	**0.017**	-0.27	0.690	0.57	0.390
	(-0.04, 1.69)		(0.25, 2.48)		(-1.60, 1.06)		(-0.74, 1.88)	
Living alone	-0.11	0.877	-0.59	0.517	2.85	**0.009**	0.22	0.837
	(-1.51, 1.29)		(-2.37, 1.20)		(0.71, 4.99)		(-1. 92, 2.37)	
Living together	Ref		Ref		Ref		Ref	
**Clinical**								
Radius patients	-0.23	0.744	-0.51	0.574	-1.24	0.258	0.53	0.619
	(-1.62, 1.16)		(-0.56, 0.57)		(-3.39, 0.91)		(-1.58, 2.65)	
Controls	Ref		Ref		Ref		Ref	
Osteopenia**	-1.22	0.143	-1.08	0.301	-0.76	0.547	-2.41	0.051
	(-2.85, 0.41)		(-3.14, 0.97)		(-3.21, 1.71)		(-4.82, 0.01)	
Osteoporosis**	-1.59	0.118	-0.69	0.597	0.98	0.531	-2.35	0.131
	(-3.58, 0.41)		(-3.26, 1.88)		(-2.1 0, 4.05)		(-5.39, 0.70)	
Normal BMD	Ref		Ref		Ref		Ref	
BMI	-0.20	**0.019**	-0.15	0.178	-0.06	0.630	-0.25	0.054
	(-0.37, -0.03)		(-0.36, 0.07)		(-0.19, 0.32)		(-0.51, -0.01)	
Co-morbidity	-0.01	0.965	-0.77	0.112	-0.92	0.075	-0.92	0.077
	(-0.67, 0.64)		(-1.71, 0.18)		(-1.24, 1.97)		(-1.94, 0.10)	
≥ 1 fall in the last year	-1.04 (-2.39, 0.32)	0.132	-1.13 (-2.86, 0.60)	0.201	0.67 (-1.42, 2.76)	0.527	-0.06 (-2.10, 1.98)	0.953
No fall	Ref		Ref		Ref		Ref	
**HRQOL/GQOL**								
MHAQ incl	-0.31	**<0.001**						
	(-0.41, 0.14)							
PCS incl			-0.39 (-0.50, -0.28)	**<0.001**				
MCS incl					-0.36 (-0.47, -0.25)	**<0.001**		
QOLS incl							-0.25 (-0.36, -0.13)	**<0.001**
**R_2 _adj**	15.5%		17.7%		16.9%		11.0%	

Regression analyses of demographics, clinical characteristics, and rescaled MHAQ at inclusion of changes in MHAQ/SF-36 at inclusion on change in SF-36/QOLS at inclusion of changes in QOLS. Adjusted unstandardized regression coefficients, 95% CI, p values.

P-values marked with bold indicate statistically significance.

* Age in decades.

** Osteopenia/osteoporosis at total hip and/or spine L2-L4.

BMD = bone mineral density, BMI = body mass index, MHAQ = Mean total MHAQ (rescaled MHAQ, range 0–100, where 100 means favourable perception of ability to perform activities of daily living), PCS = physical component summary, MCS = mental component summary (range 0 – 100, where 100 means perfect health), QOLS = quality of life scale (range 16 – 112), where 112 means high GQOL

Interaction terms between pairs of each independent variable and the patients/controls dichotomy (tested one pair at a time, with main effects retained) revealed no significantly different effects between the patients with wrist fracture and the controls in the regression analyses.

## Discussion

A low-energy distal radius fracture was not identified as a significant predictor of worsened HRQOL or GQOL one year after fracture, and the changes in HRQOL and GQOL in patients with low-energy distal radius fracture seem to be within the normal range of variation in an elderly population. Only a small proportion of the patients with a distal radius fracture did not attain their prefracture arm functions one year after fracture, even when using a modest change or slightly decreased function to identify this group [35].

The proportion of patients with distal radius fracture who did not attain their pre-fracture level of physical health (as assessed by the SF-36) was larger than the proportion of patients who did not attain their pre-fracture arm functions (as assessed by the MHAQ). The same pattern of changes was reported by the controls. This might reflect the fact that SF-36, which was used to measure physical health, is comprised of different items or skills than those included in MHAQ, e.g. walking long distances and doing outdoor activities [18,23,24]. Such skills deteriorate with aging [36], and the changes in physical health might therefore reflect normal changes or be within the normal range of variation appearing in this age-group.

Despite high levels of GQOL, significantly worsened GQOL was found in both fracture and control group. However, it should be added that separate analyses using comparative data from a nationwide sample indicate that the mean GQOL scores reported at inclusion as well as at one year follow-up by the patients and the controls in the present study were significantly higher than the scores observed in the general Norwegian population [37]. The same pattern of decrease in GQOL over a one year period has been observed within other patient groups [38,39]. Furthermore, it might be that those patients and controls who agreed to participate did so at a point in time when their GQOL was better than their own typical (long-term) level, thus creating a "regression to mean" effect when one year later that had returned to their usual level of GQOL [14]. The decreased GQOL scores in both patients with distal radius fracture and controls might be explained by the influence of non-medical factors such as characteristics of the individual and the environment like coping and retirement [40-45], – factors which have not been focused in this study.

Known correlates/covariates of HRQOL and GQOL like demographical and clinical variables could only to a limited extent predict changes in HRQOL and GQOL. Different methods of fracture treatment might have explained some of the changes in HRQOL. However, the methods of treatment used in each case have unfortunately not been included in the study, and Handoll et al [16] showed insufficient evidence to confirm differences in functional outcome between plaster cast and external fixation treatment [16]. Moreover, several studies have shown that diseases or injuries (e.g. a wrist fracture), and HRQOL and GQOL have bidirectional relationships, though all are influenced by characteristics of the individual and the environment [40,43-46]. Furthermore, the studies have shown that characteristics of the individuals and the environment influence HRQOL and GQOL differently, and non-medical factors seem to influence GQOL more than HRQOL [40,43-45]. In line with earlier studies, low education seems to be a predictor of changes in self-reported health outcomes [13,47]. However, in general it seems difficult to give a plausible substantive explanation of the worsening in HRQOL and GQOL observed in our elderly study-population.

The population-based and unselected group of patients with distal radius fracture along with matched controls may be seen as strengths of the present study. However, this study has some limitations, which should be considered when interpreting the findings. When included in the study briefly after the fracture had occurred, the patients were asked to evaluate their "pre-fracture" HRQOL and GQOL. Changes in health, such as having experienced a fracture, might cause a shift in how the patients perceived their prefracture HRQOL and GQOL (selective reporting bias and response shift) [48]. On the other hand, patients who have experienced a recent change in health have been found to be more likely to give accurate responses [33,49,50]. The patients were asked to think of the period before the fracture, and in most of the patients, HRQOL and GQOL were assessed within the first two weeks after the fracture. It seems unlikely that the patients at this point were unable to accurately recall their HRQOL and GQOL immediately before and at the time of the fracture.

We probably reached the healthiest patients with distal radius fracture in our region in our study. The patients unwilling to participate and the excluded patients were significantly older and probably less healthy than the patients included in the study [51]. Our finding might therefore be applied to other relatively healthy patients with distal radius fracture aged 50 years and older.

## Conclusion

Patients with a low-energy distal radius fracture seem to manage well one year after the fracture, and the distal radius fracture is not an independent predictor of worsened HRQOL or GQOL. This might be attributed to the fracture being experienced as a minor trauma and to the successful treatment. The proportion of patients with distal radius fracture who did not attain their pre-fracture HRQOL and GQOL seems to be comparable with the normal range of variation in this age-group. A low-energy distal radius fracture might not be considered a substantial trauma with consequences in the long run, and hence not calling for additional health care efforts.

## Abbreviations

ART: antiresorptive treatment; BMD: bone mineral density; BMI: body mass index; DXA: dual-energy X-ray absorptiometry; GLM: General Linear Model; GQOL: global quality of life; HRQOL: health related quality of life; MCS: mental component summary; MHAQ: Modified Health Assessment Questionnaire; PCS: physical component summary; SF-36: Short Form-36; s-score: standard difference score; QOLS: The Quality of Life Scale; WHO: World Health Organization.

## Competing interests

The authors declare that they have no competing interests.

## Authors' contributions

GR initiated this paper as a part of a larger study of fracture patients, collected and analyzed the data and wrote the manuscript. GH was the principal investigator for the study and supervised GR. AM supervised GR during the analyzes and drafting of the paper. TM provided statistical advice. AKW supervised GR during the analyzes and drafting of the paper. All authors critiqued revisions of the paper and approved the final manuscript

## Pre-publication history

The pre-publication history for this paper can be accessed here:


